# Automated recognition of functioning, activity and participation in COVID-19 from electronic patient records by natural language processing: a proof- of- concept

**DOI:** 10.1080/07853890.2021.2025418

**Published:** 2022-01-18

**Authors:** Carel G. M. Meskers, Sabina van der Veen, Jenia Kim, Caroline J. W. Meskers, Quirine T. S. Smit, Stella Verkijk, Edwin Geleijn, Guy A. M. Widdershoven, Piek T. J. M. Vossen, Marike van der Leeden

**Affiliations:** a Department of Rehabilitation Medicine, Amsterdam University Medical Centers, Amsterdam Movement Sciences, Amsterdam, The Netherlands; b Department of Ethics, Law and Humanities, Amsterdam University Medical Centers, Amsterdam, The Netherlands; c Computational Lexicology and Terminology Lab, Faculty of Humanities, Vrije Universiteit Amsterdam, Amsterdam, The Netherlands

**Keywords:** COVID-19, rehabilitation, ICF, functioning, electronic health record, natural language processing

## Abstract

**Purpose:**

To address the feasibility, reliability and internal validity of natural language processing (NLP) for automated functional assessment of hospitalised COVID-19 patients in key International Classification of Functioning, Disability and Health (ICF) categories and levels from unstructured text in electronic health records (EHR) from a large teaching hospital.

**Materials and methods:**

Eight human annotators assigned four ICF categories to relevant sentences: Emotional functions, Exercise tolerance, Walking and Moving, Work and Employment and their ICF levels (Functional Ambulation Categories for Walking and Moving, metabolic equivalents for Exercise tolerance). A linguistic neural network-based model was trained on 80% of the annotated sentences; inter-annotator agreement (IAA, Cohen’s kappa), a weighted score of precision and recall (F1) and RMSE for level detection were assessed for the remaining 20%.

**Results:**

In total 4112 sentences of non-COVID-19 and 1061 of COVID-19 patients were annotated. Average IAA was 0.81; F1 scores were 0.7 for Walking and Moving and Emotional functions; RMSE for Walking and Moving (5- level scale) was 1.17 for COVID-19 patients.

**Conclusion:**

Using a limited amount of annotated EHR sentences, a proof-of-concept was obtained for automated functional assessment of COVID-19 patients in ICF categories and levels. This allows for instantaneous assessment of the functional consequences of new diseases like COVID-19 for large numbers of patients.

**Key messages**
1. Hospitalised Covid-19 survivors may persistently suffer from low physical and mental functioning and a reduction in overall quality of life requiring appropriate and personalised rehabilitation strategies.
2. For this, assessment of functioning within multiple domains and categories of the International Classification of Function is required, which is cumbersome using structured data.
3. We show a proof-of-concept using Natural Language Processing techniques to automatically derive the aforementioned information from free-text notes within the Electronic Health Record of a large academic teaching hospital.

## Introduction

The new disease COVID-19 may profoundly affect long-term functioning. Recent studies suggest that after hospitalisation, patients may suffer from a reduction in overall quality of life with persistent low physical and mental functioning [1–5]. Almost 80% of hospitalised patients report at least one persistent symptom at 6 months [6] to one year after symptom onset [7,8]. Impairments in multiple categories within the functions, activity- and participation domain of the International Classification of Functioning, Disability and Health (ICF) [9] underline the need for appropriate and personalised rehabilitation during hospitalisation as well as post-discharge [10]. Deployment of such rehabilitation requires insight into the course and predictors of the level of functions, activities and participation over multiple ICF categories. This information is usually derived from structured questionnaires and measurements or could be manually extracted from health records [11] which is cumbersome and of which results only become available afterwards for relatively small numbers of patients.

Instantaneous assessment in large groups of patients is possible by recent developments in the field of artificial intelligence by analysing the rich and diverse information available in the Electronic Health Records (EHR), mainly encompassing written notes from health care professionals [12]. Relevant information in these unstructured rather than structured text data can be recognised and harvested automatically using computational modelling (natural language processing, NLP) [13,14]. Free text descriptions exhibit large variation and ambiguity that are difficult to handle by such search tools with consequences for precision and recall [15]. Mobility function information was shown to be reliably captured from physical therapy clinical notes using human annotation [16]. NLP coding was subsequently shown to be able to link narrative observations of mobility status to standardised ICF codes [17]. Recently, a comprehensive analysis of mobility functioning information, including annotation, machine sequence labelling and quality control was described [18]. However, automated functional assessment of COVID-19 patients requires labelling of multiple ICF categories and their levels within written EHR notes from different involved healthcare professionals. Such reports contain very diverse information on the functioning of patients, formulated using a mixture of expert and laymen expressions. Detecting ICF categories in such notes is challenging due to the large variation and ambiguity in a linguistic sense.

The aim of the present study was to describe a methodology for and determine the feasibility, reliability and internal validity of using NLP for the automated assessment of the level of functioning, according to different key categories of the ICF. Unstructured data was obtained from the EHR’s of non-COVID-19 and COVID-19 in- and outpatients, exhibiting large variation and ambiguity in textual structure and expressions.

## Materials and methods

### Initial dataset

Clinical notes were obtained from the electronic health records (EHR) of the Amsterdam University Medical Centres for the years 2017 to June 2020.

### Ethics

An experienced hospital privacy officer was consulted to determine the legal, privacy, and ethical prerequisites. The data were stored on a secure server at the hospital. Study approval was obtained by the Medical Ethical committee of Amsterdam University Medical Centre (ID number 2020.277).

### General approach

A standard machine learning protocol was adopted, encompassing the following steps: (1) determination of COVID-19 relevant ICF categories and levels; (2) selection of sample texts from the initial dataset; (3) human annotation of the selected texts with ICF categories and levels; (4) construction of the classification model; (5) training and optimising the classification model (ICF classifier) for the ICF categories and levels; (6) calculating the performance of the ICF classifier.

### Determination of ICF categories and levels

Potentially relevant ICF categories for COVID-19 patients were initially selected from the literature. A final choice was made based on consensus meetings within a multidisciplinary ad-hoc expert panel in an iterative way. Based on the identified literature, an initial choice for ICF categories relevant for COVID-19, four relevant ICF categories were selected to be highly impacted by this novel disease [19,20]: Emotional functions (b152), Exercise tolerance functions (b455), Walking and Moving (d450–d469), Work and Employment (d840–859, Table 1). Levels were aligned with existing scales, i.e. the Functional Ambulation Categories (FAC) and metabolic equivalents (METs), else according to the ICF.

**Table 1.** COVID-19 relevant ICF categories and levels.

	Category			
Level	Walking and Moving	Exercise tolerance	Emotional functions	Work and employment
0	No ability. The patient cannot walk or needs help from two or more people to walk or walks in a walkway.	Equivalent to 0 ≤ MET < 1. Only lying activities can be sustained physically	(Very) anxious, gloomy, angry, sad, unstable, tense, etc. and/or very often/quickly occurring mood switches.	The patient is not able to work/study.
1	Dependent (first degree). The patient needs continuous solid support from a person to bear weight and maintain balance.	Equivalent to 1 ≤ MET < 2. Activities while lying and sitting can be sustained.	A little anxious, sad, angry, sad, etc. and/or often occurring mood switches.	Very limited ability to work/study
2	Dependent (second degree). The patient needs continuous or intermittent assistance in maintaining balance or coordination.	Equivalent to 2 ≤ MET < 3. Walking at a slow to moderate pace can be physically sustained, as well as shopping and small household tasks.	Neutral.	The patient is able to work/study for about 50% of their full capacity when healthy, or the patient is only able to work/study at home and is not able to go to school/the office.
3	Supervision. The patient needs supervision from a person for safety and needs at most verbal guidance while walking. However, the patient does not need physical contact to walk.	Equivalent to 3 ≤ MET < 4. Walking and/or cycling at a normal pace, gardening and exercises without equipment are possible.	A little cheerful, positive, happy, content, stable, etc.	The patient is able to work/study at almost their full capacity when healthy.
4	Independent (limited). The patient can walk independently on a flat surface, but cannot safely climb stairs, climb slopes, or walk on uneven surfaces.	Equivalent to 4 ≤ MET ≤ 6. Cycling and/or walking at a high pace, considerable exercises such as cycling from 16 km/h and heavy housework can be physically sustained.	(Very) cheerful, positive, happy, content, stable, etc.	The patient is able to work/study at full capacity when healthy.
5	Independent. The patient can walk independently on a flat surface, on uneven surfaces, on slopes and can climb stairs.	Equivalent to MET > 6. Jogging, strenuous exercise, running, climbing stairs quickly, and sports can be sustained.	–	–

### Selection of sample texts from the initial dataset

A first sample was taken from the 2017 to 2018 records, which encompassed non-COVID-19 data only. A second sample encompassed COVID-19 patient notes only and was taken from the 2020 records using the tag for COVID-19 diagnosis that was provided in the patient data. To enhance the number of positive examples, stratified sampling was applied to the data using keywords that were associated with each of the selected ICF categories. Only the notes with keyword matches were considered and randomised sampling was applied within these to get equal coverage across the four target ICF categories. Finally, 3996 notes were selected from non-COVID-19 patients and 1583 from patients diagnosed with COVID-19.

### Human annotation of the selected texts with ICF categories and levels

Eight native Dutch speaking medical, PT or dental students were recruited for the annotation process. The annotation was supported by two students with a language-technology background and trained in-text annotation tasks. Annotation guidelines were created within the iterative process of defining the ICF categories and levels with criteria to check their applicability and examples to illustrate the process. Guidelines were applied to a subset of 30 documents shared by all annotators, collecting problematic examples through a shared spreadsheet and discussing these examples in a joint meeting. Problematic examples were documented in the new versions of the guidelines. This process was repeated until no major issues were detected and the consensus was reached. Once the guideline was finalised, annotators were provided with separate random batches from the total set of 3996 patient notes. The notes were loaded one by one in the annotation environment InCepTion (The Ubiquitous Knowledge Processing (UKP) Lab at the Department of Computer Science, Technische Universität Darmstadt, https://inception-project.github.io) from the secured server. The text was presented in separate sentences, line-by-line. Annotators were instructed to annotate each line separately for the categories and the levels by marking the relevant words in the sentence. Sentences without annotation were considered as negative examples for which no ICF category applied. The annotators first completed the non-COVID-19 batch, after which they continued with the COVID-19 batch.

### Construction of the classification model (ICF classifier)

The classification of sentences was split into two separate tasks: (1) classifying the ICF categories and (2) classifying the level for the assigned category. The former was treated as a multilabel classification task whereas the latter was considered as a regression task. Due to the computing limitation of the server where the data was hosted (CPU only), we used a support vector machine (SVM) model for the multilabel classifier (task 1) and a logistic regression model for the level labelling (task 2), both as provided in the Scikit-learn package, version 0.24 (https://scikit-learn.org/stable). Sentences were transformed into vector representations using BERTje [21]; a version of the pre-trained neural network BERT (Bidirectional Encoder Representations from Transformers [22]), specifically made for Dutch text.

### Training and testing of the ICF classifier

For each ICF category, a classifier was trained on 80% and tested on 20% of the data. Since most sentences in the notes did not receive a label, we down-sampled the negative examples. Empirical testing for the COVID-19 data showed that down sampling to 12.5% gave the best results. For the non-COVID-19 data down sampling to 25% gave the best results. To establish the impact of the size of the training data on performance, the COVID-19 data were cut up into smaller parts (20%, 40%, 60% and 80% of the complete COVID-19 training data set). These models were trained and tested on COVID-19 data and were evaluated on the sentence level.

### Calculating the performance of the ICF classifier

Annotators were paired in two teams of two to establish the Inter-Annotator-Agreement (IAA). IAA was calculated using Cohens’ kappa and Krippendorf’s alpha. Model performance regarding category identification was calculated by recall (True Positives/(True Positives + False Negatives) and precision (True Positives/(True Positives + False Positives); F1 represents the harmonic average of precision and recall: F1 = 2 × ((Precision × Recall)/(Precision + Recall)). Model performance regarding level identification was calculated by Mean Squared Error (MSE), Mean Absolute Error (MAE) and Root Mean Squared Error (RMSE).

## Results

In total, 4112 sentences of non-COVID-19 and 1061 sentences of COVID-19 notes were annotated (5173 sentences in total). IAA scores for annotator pair one reached a Cohen-Kappa of 0.76 and a Krippendorff-Alpha of 0.61. Pair two reached a Cohen-Kappa of 0.86 and a Krippendorff-Alpha of 0.59. The average Cohen-Kappa score was 0.81 and the average Krippendorff-Alpha score was 0.6.

**Figure 1.**
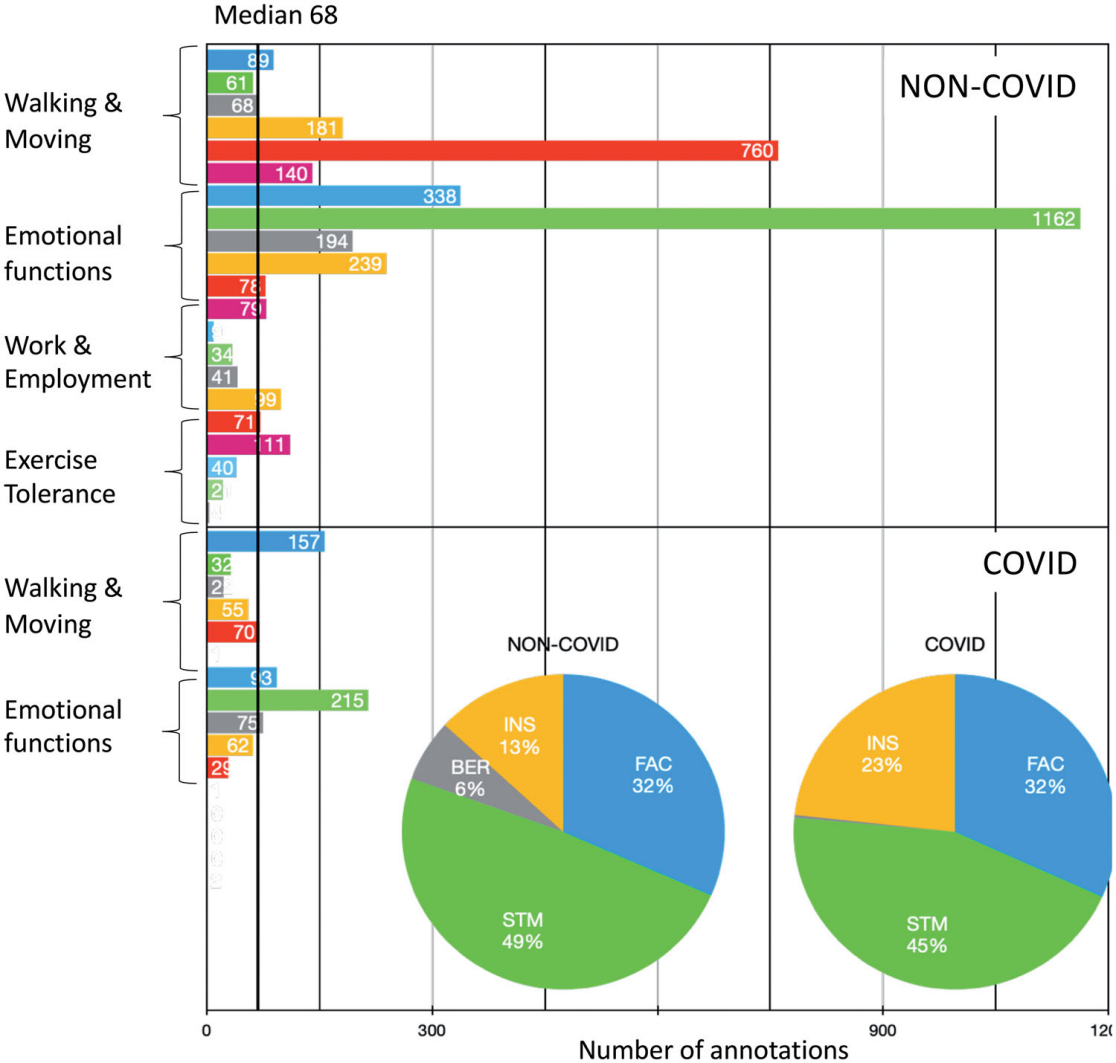
Overview of the annotation data divided into non-COVID-19 and COVID-19 notes for each annotated ICF category. Each category is differentiated in levels (0–4 and in the case of Walking & Moving and Exercise Tolerance 0–5).

The distribution of the annotations is shown in Figure 1. The total amount of annotations of non-COVID-19 was almost four times the amount of COVID-19 data, with a similar distribution of the categories except for Work and Employment.

**Figure 2.**
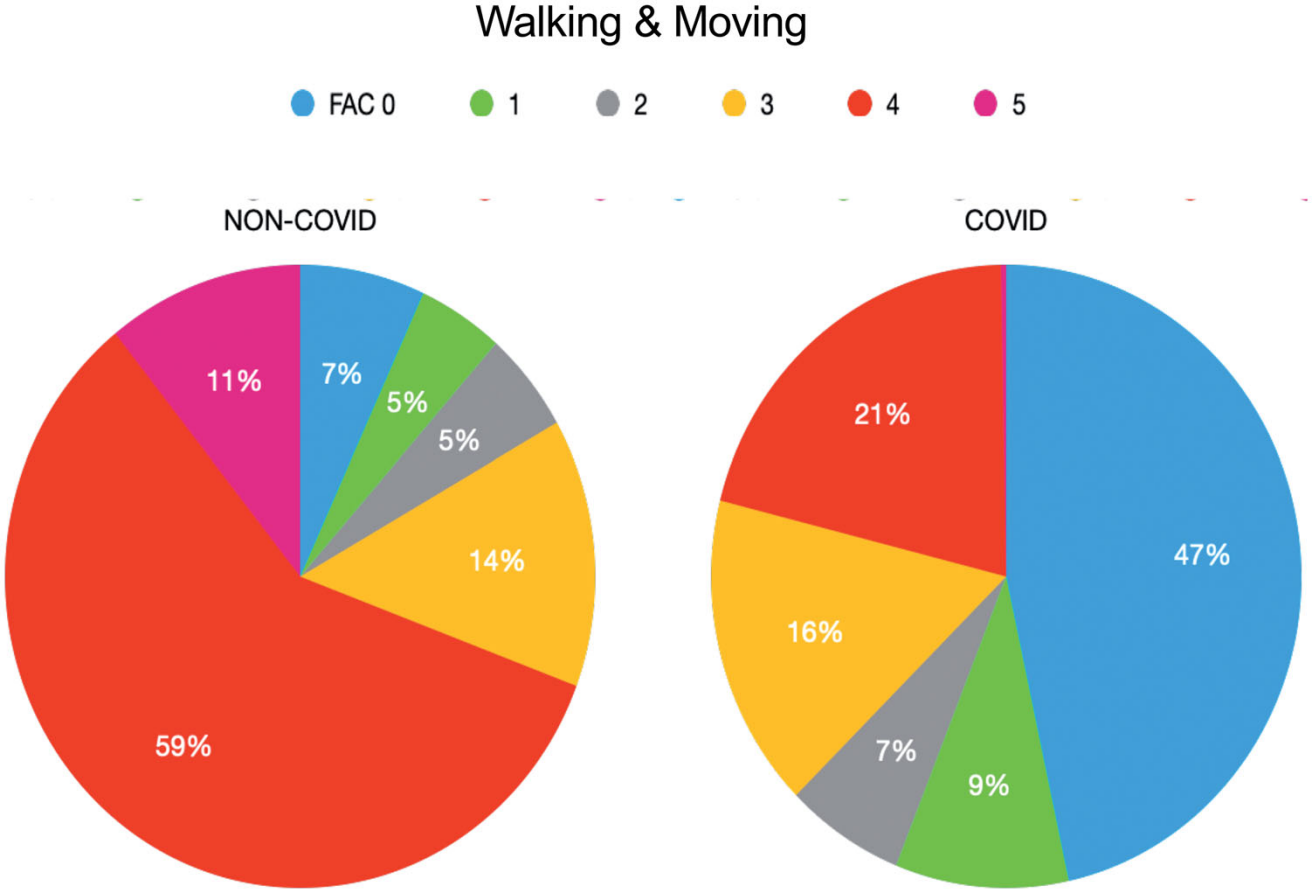
Distribution of level annotations for Walking & Moving (FAC score) across COVID-19 and non-COVID-19 data.

The distribution of the levels for the COVID-19 and non-COVID-19 cohorts is shown in Figure 2. For the category Walking and Moving, level 4 was dominant for non-COVID-19 notes (59% versus 21% COVID-19) and level 0 for COVID-19 notes (47% versus 7% non-COVID-19).

As shown in Table 2, an F1 score of 0.7 was obtained for the classification of Emotional functions in non-COVID-19 data and scores close to 0.7 for the classification of Walking and Moving in both non-COVID-19 and COVID-19 data. Classification of Exercise tolerance functions showed low performance and annotations of the category Work and Employment were too sparse in this specific dataset of patients.

**Table 2.** Performance of the ICF category classification.

	Non-COVID-19 test set			COVID-19 test set		
	*P*	*R*	F1	*P*	*R*	F1
**Walking & moving**	**0.723**	**0.661**	**0.691**	**0.733**	**0.629**	**0.677**
Emotional functions	0.771	0.646	0.703	0.545	0.476	0.508
Exercise tolerance	0.676	0.247	0.356	0.667	0.240	0.353

Work and Employment were omitted because of the lack of data in the COVID-19 annotations. P: precision; R: recall; F1: harmonic mean. Models NOT trained on combined datasets (training data of COVID-19 and non-COVID-19) are in bold. If a score is in bold, it means that it was trained only on the type of data that it was tested on (trained on non-COVID-19 and tested on non-COVID-19, for example). All the best models are evaluated at the note level.

Table 3 shows the performance of the regression models in terms of Mean Squared Error (MSE), Mean Absolute Error (MAE) and Root Mean Squared Error (RMSE) for prediction of the levels of Walking and Moving and Emotional functions for non-COVID-19 and COVID-19 patients respectively. Combining COVID-19 and non-COVID-19 data improved the results. The only exception to this was the level prediction for Walking and Moving” with an RMSE of 1.94 compared to 1.17 in COVID-19 data only.

**Table 3.** Best models regression analysis for each relevant scoring level.

	Non-COVID-19			COVID-19		
	MSE	MAE	RMSE	MSE	MAE	RMSE
Walking & moving	1.65	0.81	1.28	**1.37**	**0.91**	**1.17**
Emotional functions	0.44	0.47	0.66	0.67	0.64	0.82

Exercise Tolerance and Work and Employment were omitted because of the lack of data in the COVID-19 annotations.

MSE: Mean Squared Error; MAE: Mean Absolute Error; RMSE: Root Mean Squared Error.

**Figure 3.**
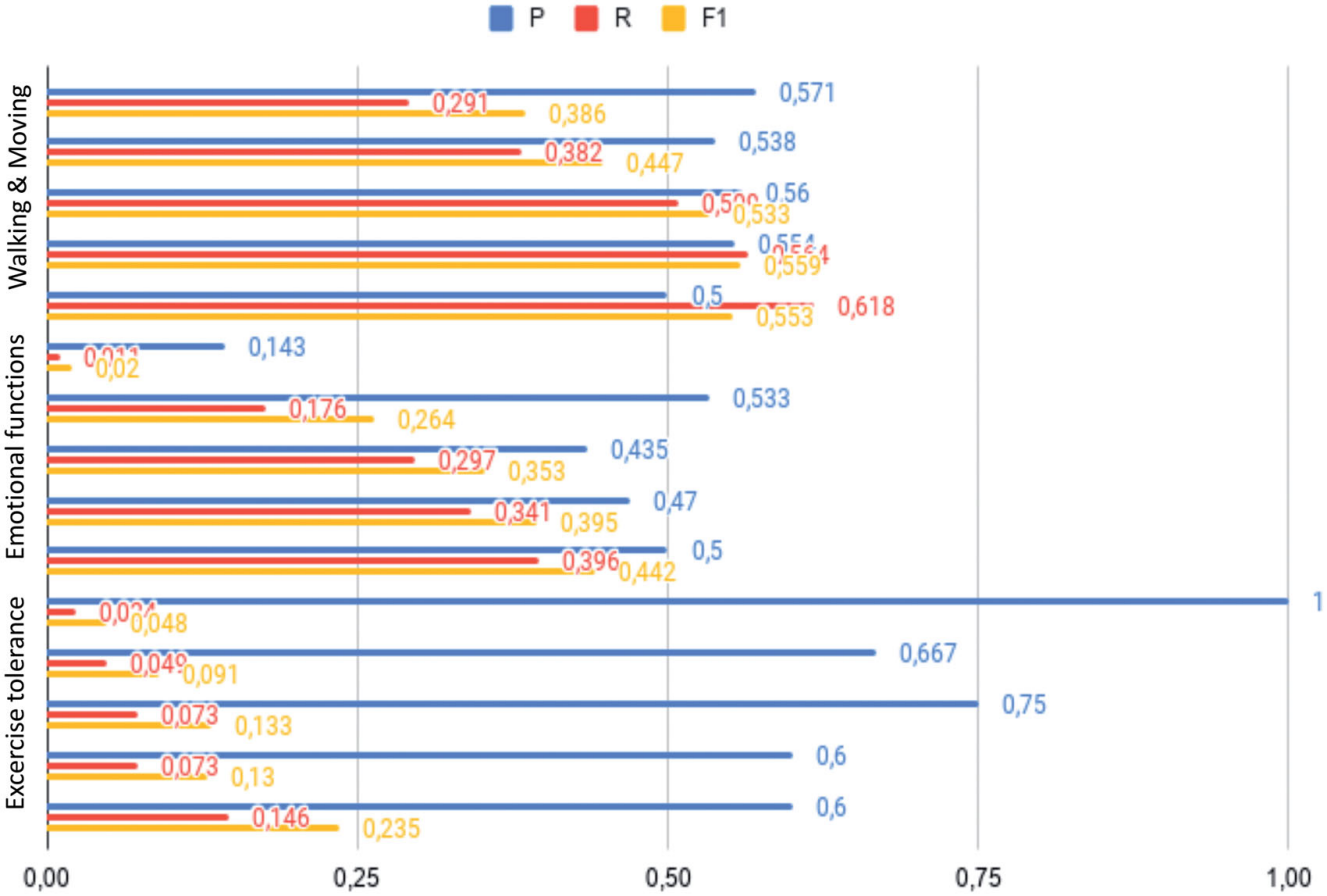
Impact of the size of the training data on performance on COVID-19 data. Per category, grouped bars from top to bottom show the magnitude of precision (P), recall (R) and their harmonic mean (F1) as a function of increasing sample size.

The results of the impact of the size of the training dataset on COVID data are shown in Figure 3: per category, grouped bars from top to bottom show the magnitude of precision (P), recall (R) and F1 as a function of increasing size. Figure 3 shows that recall is always increasing as the data size increases, and precision is not decreasing linearly.

## Discussion

The present paper provides a proof-of-concept for the automated recognition of multiple ICF categories and their levels from written notes by different health care professionals in EHRs for non-COVID-19 and -COVID-19 patients using natural language processing. Four ICF categories, Emotional functions, Exercise tolerance, Walking and Moving and Work and Employment were considered important for COVID-19 patients; levels were chosen either based on existing scores i.e. functional ambulation categories, metabolic equivalents or according to the ICF. Using an iterative annotation procedure, Cohen-Kappa scores of the inter-annotator agreement were higher than 0.8 and, with a limited amount of data, F1 scores up to 0.7 for two ICF categories were reached.

As far as we know, this is for the first time, a comprehensive procedure is described for the automated detection of multiple ICF categories and assignment of their levels from EHR free text notes based on an iterative annotation process. Facing the large variation and ambiguity of free-text notes, an iterative annotation process is a prerequisite for reaching sufficient accuracy. Agreement of human coders was previously compared with automated NLP selection of ICF code and assignment of performance- and capacity qualifiers in rehabilitation discharge notes [23]. Low performance of the NLP algorithm in the sense of precision and recall in recognising conceptual text requiring interpretation shows the need for robust annotation. Robust annotation guidelines were developed based on the mobility domain of the ICF [16]. Recently, using a small set of selected clinical narratives from physical therapy encounters, an F1 of 0.84 was reported for the classification of the mobility domain using similar NLP techniques [17,18]. Our work differs from previous work in several aspects. First, rather than targeted clinical narratives, we used more diverse and rich notes that covered a larger variety of information coming from a large variety of health professionals. Using diverse and rich notes made our task more difficult and complex, as there was more variation and ambiguity of the language used. Secondly, we considered multiple domains, among which non-clinical domains such as Emotional function, whereas former authors only used specific classifications within a single domain. Thirdly, while former authors used existing English contextualised language models trained on PubMed (BioBERT) [24] or on PubMed with EHR fine-tuning (clinicalBERT) [25], we used a general Dutch language model not adapted to clinical language. Fourthly, given the specific target on Mobility, former authors were able to differentiate the task into specific classification tasks for detecting expressions for actions, assistance and quantification. In our case, the ICF domains were less strictly defined and full sentence classification was required to capture their semantics. We demonstrated that our performance (F1 score of 0.70) is nevertheless competitive with previous work given the small training set we used.

Varying the size of the training dataset shows that increasing the amount of annotated data will further increase the performance of the model in recognising ICF categories. Low recall of the ICF category Exercise tolerance may be explained by its wide range of lexical representations which were not captured to a sufficient degree by the current annotation efforts. For Work and Employment, a low number of annotated sentences prevented further analysis. This category is assumed to be more relevant after discharge of COVID-19 patients which data was only sparsely available in our dataset.

Further improvements in performance may be reached by using different and/or optimised text modelling techniques. We are currently developing a customised language model from a large database of clinical notes that is not specific to any type of patient. This model is based on the state-of-the-art Transformer model RoBERTa [26] which was specifically built to improve the BERT model, which BioBERT and clinicalBERT are based on. The expectation is that this neural network can recognise the difference between e.g. the two mentions of “walk” in the sentences “The patient was walked through the treatment protocol” and “The patient was able to go for a 10- minute walk,” which is important for our investigation. We currently encoded each sentence in the data set by the mean function over the tokens in the last four layers of the transformer model. This resulted in a one-dimensional tensor with 768 dimensions that is given to a support vector machine (SVM) classifier. With our customised language model, we will be able to directly fine-tune the model for classification on top of all 12 layers with 1014 dimensions [27]. Based on the literature, we expect this to give a further boost in performance.

The importance of annotating a specific target population can be observed by the different handling of non-COVID-19 versus COVID-19 patients. For instance, when predicting the category Walking and Moving and its levels using COVID-19 data as test data, it was best to only use COVID-19 data during the training phase, even though the batch of non-COVID-19 data was bigger with a potential of better performance. This indicates that the population of non-COVID-19 patients is not representative of COVID-19 patients.

When considering the scores of level assignments, it is important to take into consideration that Walking and Moving has 6 levels while Emotional functions has 5. A difference of 1.17–1.28 on a scale of 6 is a good score, as is a deviation of 0.66–0.82 on a scale of 5. The increase of RMSE when combining non-COVID and COVID data can be explained by the big difference in level data across non-COVID-19: level annotations of Walking and Moving into FAC-scores are very different for COVID-19 and non-COVID-19. The regression results confirm this difference in the data. This means it is not fruitful to use non-COVID-19 data when training a model to predict COVID-19 data, as a machine learning model may be overfitted on a class that it comes across most frequently.

Our results demonstrate the potential of computational language modelling to classify ICF categories and their levels. Extension to other relevant ICF categories and further improvement of the current ICF classifiers are needed by expanding and improving the annotation guidelines and annotating more clinical notes. The developed ICF classifiers can be used to automatically analyse large amounts of EHR data, with a specific focus on the course and prediction of functioning after COVID-19 infection. These data can subsequently be combined with the structured patient- and treatment-specific data as stored within EHRs or collected in any other way, using functional status as an outcome variable in any COVID-19 related research.

### Study strength & limitations

A rigorous annotation procedure was adopted in a large data-set of EHR notes from both non-COVID and COVID-19 patients. Due to the specific sample of hospitalised patients, the low prevalence of category Work & Employment in the COVID-19 sample prevented further analysis; extension with data from home-care providers such as general practitioners and physiotherapists is warranted.

## Conclusions

A proof-of-concept is obtained by demonstrating feasibility, reliability and internal validity for the automated recognition of multiple ICF categories and their levels from written notes in a hospital EHR by different health care professionals for non-COVID and -COVID-19 patients using natural language processing.

## Data Availability

The data that support the findings of this study are available on request from the corresponding author, CGM. The data are not publicly available due to containing information that could compromise the privacy of research participants.

## Abbreviations

EHR: Electronic Health Record
BERT: Bidirectional Encoder Representations from Transformers
COVID: Corona Virus Disease
IAA: Inter-Annotator Agreement
ICF: International Classification of Function
FAC: Functional Ambulation Categories
MET: Metabolic equivalent
MAE: Mean Absolute Error
MSE: Mean Squared Error
NLP: Natural Language Processing
RMSE: Root Mean Square Error
SVM: Support Vector Machine model.
